# Impact response of a Timoshenko-type viscoelastic beam considering the extension of its middle surface

**DOI:** 10.1186/s40064-016-1751-2

**Published:** 2016-02-27

**Authors:** Yury A. Rossikhin, Marina V. Shitikova, Maria Guadalupe Estrada Meza

**Affiliations:** Research Center on Dynamics of Solids, Voronezh State University of Architecture and Civil Engineering, 20-letiya Oktybrya Street 84, 394006 Voronezh, Russian Federation

**Keywords:** Impact response, Timoshenko viscoelastic beam, Rabotnov fractional exponential function

## Abstract

In the present paper, the problem of low-velocity impact of an elastic sphere against a viscoelastic Timoshenko-type beam is studied considering the extension of its middle surface. The viscoelastic features of the beam out of the contact domain are governed by the standard linear solid model with derivatives of integer order, while within the contact domain the fractional derivative standard linear solid model is utilized, in so doing rheological constants of the material in both models are the same. However the presence of the additional parameter, i.e. fractional parameter which could vary from zero to unit, allows one to vary beam’s viscosity, since the structure of the beam’s material within this zone may be damaged, resulting in the decrease of the beam material viscosity in the contact zone. Consideration for transient waves (surfaces of strong discontinuity) propagating in the target out of the contact zone via the theory of discontinuities and determination of the desired values behind the surfaces of discontinuities upto the contact domain with the help of ray series, as well as the utilization of the Hertz theory in the contact zone allow one to obtain a set of two integro-differential equations, which govern the desired values, namely: the local bearing of the target and impactor’s materials and the displacement of the beam within the contact domain.

## Background

For the first time the extension of the middle surface during impact upon a thin body has been taken into account in the problems of impact of an elastic sphere upon an elastic Timoshenko beam in (Rossikhin and Shitikova 1996) and upon an elastic Timoshenko-type thin-walled beam of open profile in (Rossikhin and Shitikova 1999). In the state-of-the-art article (Rossikhin and Shitikova 2007a) devoted to the wave theory of impact and published in 2007 in “The Shock and Vibration Digest”, the impact response of an elastic Uflyand–Mindlin plane and a Timoshenko beam was analyzed in detail.

However, in 2014, Vershinin (2014) published a conference paper, wherein the problem of impact of an elastic sphere against an elastic Timoshenko beam was considered with due account for extension of the target middle surface, in so doing the solution was based on completely wrong formulas and equations. Thus, in page 345 of (Vershinin 2014) a reader could find incorrect formula (6)

$$\begin{aligned}{}[v]\,=\,-\frac{1-\nu }{\nu }\, G_1 \,\frac{\alpha }{h_b}, \end{aligned}$$

where [*v*] is the discontinuity in the velocity of longitudinal displacement on the plane transient wave propagating along the beam during the impact process with the velocity $G_1\,=\,\sqrt{E/\rho }$, *E* is Young’s modulus, $\rho$ is the density, $\nu$ is Poisson’s ratio, $h_b$ is the beam thickness, and $\alpha$ is the local bearing the target and impactor’s materials. The above formula is valid for an elastic thin plate, but it is invalid for a beam.

However, the correct formula for the beam (which will be used further in the present paper) was derived 50 years ago in the classical work by Landau and Lifshitz (1965)

$$\begin{aligned}{}[v]=-\frac{1}{\nu }\, G_1 \,\frac{\alpha }{h_b}. \end{aligned}$$

In the same page of (p. 345 in Vershinin 2014), the equation of motion of the contact zone was presented as

$$\begin{aligned} 2N\, \frac{\partial w}{\partial z}+2Q+P(t)=\rho h \pi a^2 {\ddot{w}}, \end{aligned}$$

where *N* is the longitudinal force, *w* and *Q* are transverse displacement and force, respectively, *P*(*t*) is the contact force, *z* is the coordinate directed along the beam axis, a dot denotes the time-derivative, *a* is the radius of the contact zone, in so doing the author of (Vershinin 2014) has considered that plane transient waves (surfaces of discontinuity) propagate from the contact zone during the process of impact. However, if the contact domain is a circular disk with a volume $h\pi a^2$, then the waves travelling from a circular contact zone are diverging circles.

In order the waves propagating from the contact zone to be the plane waves, the contact domain should be a rectangular parallelepiped with the volume 2*aF*, where *F* is the cross sectional area of the beam, and the equation should have the form

$$\begin{aligned} 2N\, \frac{\partial w}{\partial z}+2Q+P(t)=2aF \rho {\ddot{w}}. \end{aligned}$$

In the same page of (p. 345 in Vershinin 2014), the final equation was presented as

$$\begin{aligned} \ddot{\alpha } +\frac{1}{m}\, P(\alpha )+c_b\, \frac{d}{d\alpha }\left( \frac{P(\alpha )}{1+e_b\alpha }\right) =0, \end{aligned}$$

where $c_b=(2\rho FG_2)^{-1}$, $G_2=\sqrt{K\mu /\rho }$, $e_b=\frac{1-\nu }{\nu }\, \frac{G_1^2}{G_2^2}\, h_b$, *K* is the shear coefficient, and $\mu$ is the shear modulus. This equation is also incorrect.

The correct equation neglecting the inertia of the contact zone (this important assumption was not mentioned in Vershinin (2014) ) has the form

$$\begin{aligned} \ddot{\alpha } +\frac{1}{m}\, P(\alpha )+c_b\, \frac{d}{d\alpha }\left( \frac{P(\alpha )}{1+e\alpha }\right) \dot{\alpha } =0 \end{aligned}$$

with the correct coefficient $e=\frac{G_1^2}{G_2^2}\, \frac{1}{\nu h_b}$.

Based on the aforesaid, the further numerical treatment presented in Vershinin (2014) occurs to be invalid.

In the present paper, we present not only the correct equation for the impact response of the elastic Timoshenko beam, but the deduction and analysis of equations describing the behaviour of a viscoelastic Timoshenko-type beam impacted by an elastic sphere are given considering the damage of the target material within the contact domain.

## Problem formulation

**Fig. 1:**
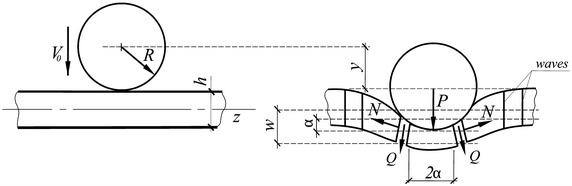
Scheme of the shock interaction of a viscoelastic beam and spherical impactor

Let an elastic sphere with the radius *R* and mass *m* move along the $y-$axis with a constant velocity $V_0$ towards a Timoshenko-type viscoelastic homogeneous isotropic beam of the width *h* (Fig. 1), viscoelastic features of which are described by the standard linear solid model with conventional derivatives. The dynamic behaviour of such a beam with due account for extension of its middle surface is described by the following set of equations:

1 $$\begin{aligned} \frac{\partial N}{\partial z}\,=\, \rho F \dot{v}, \quad \frac{\partial Q}{\partial z}=\rho F \dot{W}, \quad -\frac{\partial M}{\partial z}+Q=\rho I \dot{\Psi }, \end{aligned}$$

2 $$\begin{aligned} N \,=\, F E_\infty \left[ 1-\nu _\varepsilon \ni ^*_1(\tau _\varepsilon ) \right] \frac{\partial u}{\partial z}, \end{aligned}$$

3 $$\begin{aligned} Q\,=\, KF \mu _\infty \left[ 1-n \ni ^*_1(t_\varepsilon ) \right] \left( \frac{\partial w}{\partial z}- \psi \right) , \end{aligned}$$

4 $$\begin{aligned} M\,=\, -I E_\infty \left[ 1-\nu _\varepsilon \ni ^*_1(\tau _\varepsilon ) \right] \frac{\partial \psi }{ \partial z}, \end{aligned}$$

where *M*, *Q*, and *N* are the bending moment, the shear and longitudinal forces, respectively, *u* and *w* are longitudinal and transverse displacements, respectively, $\psi$ is the angle of rotation of the cross section around the *z*-axis, $v=\dot{u}$, $W={\dot{w}}$, $\Psi =\dot{\psi }$, *F* and *I* are the cross-sectional area and the moment of inertia with respect to the *x*-axis, respectively, $\rho$ is the density, *K* is the shear coefficient dependent on beam’s geometrical dimensions and the form of its cross section, and an overdot denotes the time derivative.

In equations (2) and (4), the operator corresponding to the Young modulus has the form

5 $$\begin{aligned} \widetilde{E}\,= \, E_\infty \left[ 1-\nu _\varepsilon \ni _1^*(\tau _\varepsilon ) \right] , \end{aligned}$$

6 $$\begin{aligned} \widetilde{E}^{-1}\,=\, E_\infty ^{-1} \left[ 1+\nu _\sigma \ni _1^*(\tau _\sigma ) \right] , \end{aligned}$$

7 $$\begin{aligned} \nu _\varepsilon\,=\, \frac{E_\infty -E_0}{E_\infty } =\frac{\Delta E}{E_\infty }, \end{aligned}$$

8 $$\begin{aligned} \ni _1^*(\tau _i ) Z(t)\,=\, \frac{1}{\tau _i } \int _0^t e^{-(t-t')/\tau _i } Z (t') dt' \quad (i=\varepsilon , \sigma ), \end{aligned}$$

9 $$\begin{aligned} \ni _1^*(t_i ) Z(t)\,= \,\frac{1}{t_i } \int _0^t e^{-(t-t')/t_i } Z (t') dt' \quad (i=\varepsilon , \sigma ), \end{aligned}$$

where *Z*(*t*) is a desired function, $E_\infty$ and $E_0$ are the non-relaxed (instantaneous modulus of elasticity, or the glassy modulus) and relaxed elastic (prolonged modulus of elasticity, or the rubbery modulus) moduli which are connected with the relaxation time $\tau _\varepsilon$ and retardation time $\tau _\sigma$ by the following relationship:

10 $$\begin{aligned} \frac{\tau _\varepsilon }{\tau _\sigma }=\frac{E_0}{E_\infty }. \end{aligned}$$

In equation (3), the operator corresponding to the shear modulus has the form

11 $$\begin{aligned} \widetilde{\mu } =\mu _\infty \left[ 1-n \ni _1^*(t_\varepsilon ) \right] , \end{aligned}$$

where $\mu _\infty$ is the nonrelaxed magnitude of the shear modulus, and *n* and $t_\varepsilon$ are yet unknown constants.

Rossikhin and Shitikova (2013, 2015) have shown that if the operator $\widetilde{E}$ is assumed to be assigned and the operator of the triaxial extension–compression ${\widetilde{K}}$, according to experimental data (Rabotnov 1966), is considered to be time-independent, i.e. ${\widetilde{K}}=K_\infty$, where $K_\infty$ is the non-relaxed module, then Poisson’s coefficient becomes a time-dependent operator

12 $$\begin{aligned} {\widetilde{\nu }} =\nu _\infty + \frac{1}{2}(1-2\nu _\infty ) \nu _\varepsilon \ni _1^*(\tau _\varepsilon ) , \end{aligned}$$

as well as the Lam$\acute{\mathrm{e}}$ constants $\lambda$ and $\mu$ take the form of the time-dependent operators

13 $$\begin{aligned} \widetilde{\lambda } =\lambda _\infty \left[ 1+n_1 \ni _1^*(t_\varepsilon ) \right] \end{aligned}$$

and $\widetilde{\mu }$ (11), respectively, where $\nu _\infty$ and $\lambda _\infty$ are the non-relaxed magnitudes of the corresponding operators, and

$$\begin{aligned} n_1 = & {} \frac{(1-2\nu _\infty )\nu _\varepsilon }{2(1+\nu _\infty )A}, \quad n = \frac{3\nu _\varepsilon }{2(1+\nu _\infty )A},\\ A = & {} \frac{2(1+\nu _\infty )+(1-2\nu _\infty )\nu _\varepsilon }{2(1+\nu _\infty )A}>1, \quad t_\varepsilon = \tau _\varepsilon A^{-1}. \end{aligned}$$

The impact occurs at $t=0$ (Fig. 1). When $t>0$, the displacement of the sphere’s center *y* could be represented in the form

14 $$\begin{aligned} y=w+\alpha , \end{aligned}$$

where $\alpha$ is the quasi-static bearing of impactor and target’s materials which is connected with the contact force by the following formula according to the generalized Hertzian law:

15 $$\begin{aligned} P(t)=\frac{4\sqrt{R}}{3}{\;\widetilde{k}}\, \alpha ^{3/2} , \end{aligned}$$

where

16 $$\begin{aligned} {\widetilde{k}}^{-1}=\frac{1-{\widetilde{\nu }} ^2_1}{\widetilde{E}_1}+\frac{1-\nu _2^2}{E_2}, \end{aligned}$$

In formula (16) indices 1 and 2 refer to the viscoelastic beam and elastic sphere, respectively, in so doing the operators $\widetilde{E}_1$ and ${\widetilde{\nu }}_1$, which act within the contact domain, differ from operators (5), (6), and (12) valid within the other parts of the target, namely:

17 $$\begin{aligned} \widetilde{E}_1\,=\, E_\infty \left[ 1-\nu _\varepsilon \ni _\gamma ^*(\tau _\varepsilon ^\gamma ) \right] \quad (0\le \gamma \le 1), \end{aligned}$$

18 $$\begin{aligned} \widetilde{E}^{-1}_1\,=\, E_\infty ^{-1} \left[ 1+\nu _\sigma \ni _\gamma ^*(\tau _\sigma ^\gamma) \right] , \end{aligned}$$

19 $$\begin{aligned} {\widetilde{\nu }}\,=\,\nu _\infty + \frac{1}{2}(1-2\nu _\infty ) \nu _\varepsilon \ni _\gamma ^*(\tau _\varepsilon ^\gamma ) , \end{aligned}$$

where all rheological constants for the fractional parameter $\gamma$ remain the same as for $\gamma =1$,

20 $$\begin{aligned}&\left( \frac{\tau _\varepsilon }{\tau _\sigma }\right) ^\gamma =\frac{E_0}{E_\infty }, \end{aligned}$$

21 $$\begin{aligned}&\ni _1^*(\tau _i ^\gamma ) Z(t)=\int _0^t \ni _\gamma \left( {\frac{t-t'}{\tau _i }}\right) Z (t') dt' \quad (i=\varepsilon , \sigma ), \end{aligned}$$

22 $$\begin{aligned}&\ni _\gamma \left( \frac{t}{\tau _i } \right) =\frac{t^{\gamma -1}}{\tau _i^\gamma } \sum \limits _{n=0}^\infty \frac{(-1)^n(t/\tau _i)^{\gamma n}}{\Gamma [\gamma (n+1)]}, \end{aligned}$$

$\Gamma [\gamma (n+1)]$ is the Gamma-function, $\ni _\gamma \left( t/\tau _i \right)$ is Rabotnov’s fractional exponential function (Rabotnov 1948) which at $\gamma =1$ goes over into the ordinary exponent, and operator $\ni _\gamma ( \tau _i )$ transforms into operator $\ni _1^* ( \tau _i )$. When $\gamma \rightarrow 0$, the function $\ni _\gamma \left( t/\tau _i \right)$ tends to the Dirac delta-function $\delta (t)$.

This distinction is connected with the fact that during the impact process there occurs decrosslinking within the domain of the contact of the beam with the impactor, resulting in more freely displacements of molecules with respect to each other, and finally in the decrease of the beam material viscosity in the contact zone (Popov et al. 2015). This circumstance allows one to describe the behaviour of the beam material within the contact domain by the standard linear solid model involving fractional derivatives, since variation in the fractional parameter (the order of the fractional derivative) enables one to control the viscosity of the beam material from its initial value at $\gamma =1$ to its vanishing at $\gamma =0$. Thus, the substitution of operators (5), (6), and (12) with operators (17)–(19), respectively, is quite reasonable.

Now the equation of motion of the sphere could be written in the form

23 $$\begin{aligned} m\ddot{y}=-P(t), \end{aligned}$$

where *P*(*t*) is defined by formula (15), while the equation of motion of the contact zone, which is considered to be rigid and is restricted by the planes $z=\pm a$ (Fig. 1)

24 $$\begin{aligned} a(t)=\sqrt{\alpha R}, \end{aligned}$$

is written as

25 $$\begin{aligned} 2N\, \frac{\partial w}{\partial z}+2Q+P(t)=2aF\rho {\ddot{w}}. \end{aligned}$$

Equations (23) and (25) are subjected to the following initial conditions

26 $$\begin{aligned} y|_{t=0}=0, \qquad \dot{y}|_{t=0}=V_0, \qquad w|_{t=0}={\dot{w}}|_{t=0}=0. \end{aligned}$$

Under the assumptions made above with respect to the contact domain, transient longitudinal and transverse waves (surfaces of strong discontinuity) propagate from the boundary of the contact zone during the impact. A certain desired function *Z*(*z*, *t*) behind the front of the wave surface is represented in terms of the ray series (Rossikhin and Shitikova 1995)

27 $$\begin{aligned} Z(z,t)=\sum \limits _{\alpha =1}^2 \sum \limits _{k=0}^\infty \frac{1}{k!} \left[ Z,_{(k)}\right] \Bigl |_{t=z/G^{(\alpha )}} \left( t-\frac{z}{G^{(\alpha )}}\right) ^k H\left( t-\frac{z}{G^{(\alpha )}}\right) , \end{aligned}$$

where $\left[ Z,_{(k)}\right] =Z,_{(k)}^+ - Z,_{(k)}^-= \left[ \partial ^k Z/ \partial t^k \right]$ are the discontinuities in the *k*-th order derivatives with respect to time *t* of the desired function *Z*(*z*, *t*) on the wave surface, the upper signs $+$ and $-$ denote that the given value is calculated immediately ahead of and behind the wave front, respectively, the index $\alpha$ labels the ordinal number of the wave, namely: $\alpha =1$ for the longitudinal wave, and $\alpha =2$ for the transverse wave, *H*(*t*) is the Heaviside function, and $G^{(\alpha )}$ is the normal velocity of the surface of discontinuity.

To determine coefficients of the ray series (27), it is necessary to differentiate the governing Eqs. (1)–(4) *k* times with respect to time, take their difference on the different sides of the wave surface $\Sigma$ and apply the condition of compatibility for the $k+1$-order discontinuities of the function *Z*, which has the following form (Rossikhin and Shitikova 1995):

28 $$\begin{aligned} G\left[ \frac{\partial Z,_{(k)}}{\partial z} \right] =-[Z,_{(k+1)}] +\frac{d [Z,_{(k)}]}{d t}, \end{aligned}$$

where *d*/*dt* is the complete time-derivative of the function $Z,_{(k)}(z,t)$ on the moving surface of discontinuity.

Since the process of impact is a transient process, then, firstly, it is possible to limit ourselves by zeroth terms of the ray series (28), and secondly, to neglect the waves reflected from the end face of the beam considering that they reach the contact zone after impactor’s rebound from the beam.

Further we shall interpret a shock wave in the beam (surface of strong discontinuity) as a layer of small thickness $\delta$, the head front of which arrives at a certain point *M* with the coordinate *z* at the moment of time *t*, while the back front of the shock layer reaches this point at the moment $t+\Delta t$. The desired values *Z*(*z*, *t*) at the point *M*, such as velocity, generalized forces and deformations, during the time increment $\Delta t$ change monotonically and uninterruptedly from the magnitude $Z^-$ to the magnitude $Z^+$, in so doing within the layer, according to the condition of compatibility (28), the relationship

29 $$\begin{aligned} \frac{\partial Z}{\partial z} \approx - G^{-1} \dot{Z} \end{aligned}$$

is fulfilled, which is the more accurate the smaller the value of $\Delta t$.

Substituting the derivatives $\partial N/\partial z$, $\partial Q/\partial z$, and $\partial M/\partial z$ in (1) with $- G^{-1} \dot{N}$, $- G^{-1} \dot{Q}$, and $- G^{-1} \dot{M}$, respectively, integrating then the resulting equations from *t* to $t+\Delta t$, and tending $\Delta t$ to zero, we find

30 $$\begin{aligned}{}[N]\, = \,-\,\rho FG [v], \quad [Q] = -\,\rho FG [W], \quad [M] = \rho I\;G [\Psi ]. \end{aligned}$$

Substituting in (2)–(4) the derivatives $\partial u/\partial z$, $\partial w/\partial z$, and $\partial \psi /\partial z$ by the expressions $-G^{-1}v$, $-G^{-1}W$, and $-G^{-1}\Psi$, respectively, and writing them at the moments *t* and $t+\Delta t$, we obtain

31 $$\begin{aligned} N^-\,= \, -FE_\infty \left[ G^{-1}v^- -\nu _\varepsilon \;\frac{1}{\tau _\varepsilon } \int _0^t e^{-\frac{t-t'}{\tau _\varepsilon }}\, G^{-1}v(t') dt' \right] , \end{aligned}$$

32 $$\begin{aligned} N^+\, = \,-FE_\infty \left[ G^{-1}v^+ -\nu _\varepsilon \;\frac{1}{\tau _\varepsilon } \int _0^{t+\Delta t} e^{-\frac{t+\Delta t-t'}{\tau _\varepsilon }}\, G^{-1}v(t') dt' \right] , \end{aligned}$$

33 $$\begin{aligned} Q^-\,= \, -KF\mu _\infty \left[ G^{-1}W^- +\psi ^- -n_\varepsilon \;\frac{1}{t_\varepsilon } \int _0^t e^{-\frac{t-t'}{t_\varepsilon }}\left( G^{-1}W(t')+\psi (t') \right) dt' \right] , \end{aligned}$$

34 $$\begin{aligned} Q^+\,= \,-KF\mu _\infty \left[ G^{-1}W^+ +\psi ^+ -n_\varepsilon \;\frac{1}{t_\varepsilon } \int _0^{t+\Delta t} e^{-\frac{t+\Delta t-t'}{t_\varepsilon }}\left( G^{-1}W(t')+\psi (t') \right) dt' \right] , \end{aligned}$$

35 $$\begin{aligned} M^- = \, IE_\infty \left[ G^{-1}\Psi ^- -\nu _\varepsilon \;\frac{1}{\tau _\varepsilon } \int _0^t e^{-\frac{t-t'}{\tau _\varepsilon }}\, G^{-1} \Psi (t') dt' \right] , \end{aligned}$$

36 $$\begin{aligned} M^+ = \, IE_\infty \left[ G^{-1}\Psi ^+ -\nu _\varepsilon \;\frac{1}{\tau _\varepsilon } \int _0^{t+\Delta t} e^{-\frac{t+\Delta t-t'}{\tau _\varepsilon }}\, G^{-1}\Psi (t') dt' \right] . \end{aligned}$$

Expanding the integrals entering in (32), (34), and (36) into the Taylor series with respect to the small parameter $\Delta t$ and limiting ourselves by the zeroth and first approximations, we have

37 $$\begin{aligned}&\int _0^{t+\Delta t}e^{-\frac{t+\Delta t-t'}{\tau _\varepsilon }}\, v(t') dt' = \int _0^{t} e^{-\frac{t-t'}{\tau _\varepsilon }}\, v(t') dt' + v(t) \Delta t \nonumber \\&\qquad \qquad \qquad \qquad \qquad -\Delta t\, \frac{1}{\tau _\varepsilon }\int _0^{t} e^{-\frac{t-t'}{\tau _\varepsilon }}\, v(t') dt', \end{aligned}$$

38 $$\begin{aligned}&\int _0^{t+\Delta t}e^{-\frac{t+\Delta t-t'}{t_\varepsilon }}\Bigl [ G^{-1}W(t')+\psi (t') \Bigr ] dt'=\int _0^{t} e^{-\frac{t-t'}{t_\varepsilon }}\left[ G^{-1}W(t')+\psi (t') \right] dt' \nonumber \\&\qquad \qquad \qquad \qquad \qquad \qquad \qquad\quad + \left[ G^{-1}W(t')+\psi (t') \right] \Delta t \nonumber \\&\qquad \qquad \qquad \qquad \qquad \qquad \qquad\quad - \Delta t\, \frac{1}{t_\varepsilon } \int _0^{t} e^{-\frac{t-t'}{t_\varepsilon }}\left[ G^{-1}W(t')+\psi (t') \right] dt' , \end{aligned}$$

39 $$\begin{aligned}&\int _0^{t+\Delta t} e^{-\frac{t+\Delta t-t'}{\tau _\varepsilon }}\, \psi (t') dt' = \int _0^{t} e^{-\frac{t-t'}{\tau _\varepsilon }}\, \psi (t') dt' +\psi (t) \Delta t \nonumber \\&\qquad \qquad \qquad \qquad \qquad - \Delta t\, \frac{1}{\tau _\varepsilon }\int _0^{t} e^{-\frac{t-t'}{\tau _\varepsilon }}\, \psi (t') dt'. \end{aligned}$$

Subtracting (31), (33), and (35), respectively, from (32), (34), and (36) with due account for (37)–(39), and tending $\Delta t$ to zero, we find

40 $$\begin{aligned}{}[N] = -\,F E_\infty G^{-1} [v], \quad [Q] = -\,KF\mu _\infty G^{-1}[W], \quad [M] = IE_\infty G^{-1} [\Psi ]. \end{aligned}$$

From relationships (30) and (40) it is possible to find the velocities of two types of transient waves:

longitudinal-flexural wave

41 $$\begin{aligned} G^{(1)}_\infty = \left( \frac{E_\infty }{\rho }\right) ^{1/2}, \end{aligned}$$

and shear wave

42 $$\begin{aligned} G^{(2)}_\infty = \left( \frac{K\mu _\infty }{\rho }\right) ^{1/2}. \end{aligned}$$

Substituting the found velocities (41) and (42) in formulae (30) and limiting, as it has been already mentioned, by the zeroth terms of the ray series, we have

43 $$\begin{aligned} N = -\,\rho F G^{(1)}_\infty v, \quad Q = -\,\rho F G^{(2)}_\infty W, \quad M = \rho I G^{(1)}_\infty \Psi . \end{aligned}$$

Note that relationships (43) differ nothing from those for an elastic beam, since at the moment of impact a viscoelastic medium behaves as an elastic medium with the unrelaxed elastic modulus.

Now it is necessary to substitute the values of *N* and *Q* defined by (43) in (25). However the governing set of two equations, (23) and (25), should involve only two unknown values, $\alpha$ and *w*, while the force *N* entering in (25) depends on the velocity *v*, as it follows from (43), and therefore *v* should be expressed in terms of $\alpha$ and *w*.

For this purpose we write the relationship for the stress tensor in a viscoelastic medium

44 $$\begin{aligned} \sigma _{ij} = \lambda _\infty \left[ 1+n_1 \ni ^*_1(t_\varepsilon ) \right] u_{l,l} \delta _{ij} +\mu _\infty \left[ 1-n \ni ^*_1(t_\varepsilon ) \right] \left( u_{i,j}+u_{j,i}\right) , \end{aligned}$$

where summation is carried out over two repeated indices, an index after a comma labels the derivative with respect to the corresponding coordinate, $\sigma _{ij}$ and $u_i$ are the stress tensor and displacement vector components, respectively, $x = x_1$, $y = x_2$, $z = x_3$, and $\delta _{ij}$ is the Kronecker symbol ($i, j = 1,2,3$).

Using the procedure applied above to deduce formulae (40), from relationship (44) we obtain

45 $$\begin{aligned}{}[\sigma _{ij}] = \lambda _\infty [u_{l,l}] \delta _{ij}+\mu _\infty \left( [u_{i,j}]+[u_{j,i}]\right) . \end{aligned}$$

Multiplying (45) sequentially by $k_ik_j$ and $s_is_j$, and neglecting the press of layers within the front of the surface of strong discontinuity in the direction of the vectors $\vec{k}$ and $\vec{s}$, i.e., considering that

46 $$\begin{aligned}{}[\sigma _{ij}] k_i k_j=[\sigma _{ij}] s_i s_j = 0, \end{aligned}$$

we have

47 $$\begin{aligned}&\lambda _\infty [u_{l,l}] +2\mu _\infty [u_{x,x}] = 0, \end{aligned}$$

48 $$\begin{aligned}&\lambda _\infty [u_{l,l}] +2\mu _\infty [u_{y,y}] = 0, \end{aligned}$$

whence it follows the equality

49 $$\begin{aligned}{}[u_{x,x}] = [u_{y,y}] . \end{aligned}$$

Considering the generalized geometric conditions of compatibility (Rossikhin and Shitikova 2007b)

50 $$\begin{aligned}{}[u_{i,j}] = \, -G^{-1} [v_i] \nu _j + [u_{i,x}] k_j+[u_{i,y}] s_j, \end{aligned}$$

51 $$\begin{aligned}{}[u_{l,l}] = \,-G^{-1} [v_{l}] \nu _l + [u_{x,x}]+[u_{y,y}], \end{aligned}$$

relationships (47) and (48) could be rewritten in the form

52 $$\begin{aligned}{}[v] = \, [v_{l}]\nu _l =2(\lambda _\infty +\mu _\infty )\lambda _\infty ^{-1} [u_{x,x}] G_\infty ^{(1)}, \end{aligned}$$

53 $$\begin{aligned}{}[v] = \, [v_{l}]\nu _l =2(\lambda _\infty +\mu _\infty )\lambda _\infty ^{-1} [u_{y,y}] G_\infty ^{(1)}. \end{aligned}$$

Since in further treatment one-term ray expansions will be used, then (52) and (53) take the form

54 $$\begin{aligned} u_{y,y}=u_{x,x} = {G_\infty ^{(1)}}^{-1}\nu _\infty v=-\nu _\infty u_{z,z}, \end{aligned}$$

or

55 $$\begin{aligned} v = \nu _\infty ^{-1}G_\infty ^{(1)} u_{y,y}=-\nu _\infty ^{-1} G_\infty ^{(1)} \, \frac{\alpha }{h}. \end{aligned}$$

Note that formula (54) has been presented in Landau and Lifshitz (1965) for elastic beams.

Thus, considering formulae (43) and (55), the force *N* could be rewritten in the form

56 $$\begin{aligned} N=\rho F {G_\infty ^{(1)}}^{2}\nu _\infty ^{-1} \alpha h^{-1}. \end{aligned}$$

Considering (56) and (24), as well as relationship

57 $$\begin{aligned} \frac{\partial w}{\partial z}=-{G_\infty ^{(2)}}^{-1} W, \end{aligned}$$

Eq. (25) could be rewritten in the form

58 $$\begin{aligned} (e\alpha +g) W + l\alpha ^{1/2} \dot{W}=P(t), \end{aligned}$$

where $$e=2\rho F {G_\infty ^{(1)}}^{2} \left( G_\infty ^{(2)} \nu _\infty h \right) ^{-1}$$, $$g=2\rho F {G_\infty ^{(2)}}$$, and $$l=2\rho F \sqrt{R}$$.

Substituting (14) in (23) yields

59 $$\begin{aligned} m \dot{W} + m \ddot{\alpha }=-P(t). \end{aligned}$$

Adding Eqs. (58) and (59), we find

60 $$\begin{aligned} m \dot{W} + m \ddot{\alpha }+ l\alpha ^{1/2} \dot{W}+(e\alpha +g) W =0. \end{aligned}$$

As it has been shown in Rossikhin and Shitikova (2013), the operator ${\widetilde{k}}$ entering in (15) has the form

61 $$\begin{aligned} {\widetilde{k}}=\left( \frac{1-{\widetilde{\nu }} _1^2}{\widetilde{E}_1}+\frac{1-\nu _2^2}{E_2}\right) ^{-1} =d^{-1}\left[ 1-e_1 \ni _\gamma ^*\left( t_1^\gamma \right) -e_2 \ni _\gamma ^*\left( t_2^\gamma \right) \right] , \end{aligned}$$

where

$$\begin{aligned} t_{1,2}^{-\gamma }= \, \frac{1}{2}\left[ \tau _\varepsilon ^{-\gamma }(1+g_1) +\tau _\sigma ^{-\gamma }(1+g_2)\right] \nonumber \\&\pm \frac{1}{2}\sqrt{\left[ \tau _\varepsilon ^{-\gamma }(1+g_1)-\tau _\sigma ^{-\gamma }(1+g_2)\right] ^2 +4 \tau _\varepsilon ^{-\gamma } \tau _\sigma ^{-\gamma }g_1g_2}\;,\\ d= & {} \frac{1-\nu _\infty ^2}{ E_\infty }+\frac{1-\nu _2^2}{E_2}, \quad g_1=\frac{(1-2\nu _\infty )^2\nu _\varepsilon }{4E_\infty }, \quad g_2=\frac{3\nu _\sigma }{4E_\infty d},\\ e_1= & {} \frac{b_2-a_2}{a_1b_2-a_2b_1}>0, \quad e_2=\frac{a_1-b_1}{a_1b_2-a_2b_1}>0,\\ a_1= & {} \frac{t_1^{-\gamma }}{t_1^{-\gamma }-\tau _\varepsilon ^{-\gamma }}>0, \quad a_2=\frac{t_2^{-\gamma }}{t_2^{-\gamma }-\tau _\varepsilon ^{-\gamma }}>0,\\ b_1= & {} \frac{t_1^{-\gamma }}{t_1^{-\gamma }-\tau _\sigma ^{-\gamma }}<0, \quad b_2=\frac{t_2^{-\gamma }}{t_2^{-\gamma }-\tau _\sigma ^{-\gamma }}>0. \end{aligned}$$

Thus Eq. (58) takes the form

62 $$\begin{aligned} m \dot{W} + m \ddot{\alpha }= & {} -k_1\Biggl [ \alpha ^{3/2}(t) -e_1\int _0^t \ni _\gamma \left( - \frac{t-t'}{t_1}\right) \alpha ^{3/2}(t')dt' \nonumber \\& \qquad \qquad \quad - e_2 \int _0^t \ni _\gamma \left( - \frac{t-t'}{t_2}\right) \alpha ^{3/2}(t')dt'\Biggr ], \end{aligned}$$

where $$k_1=\frac{4}{3}\, \sqrt{R}d^{-1}$$.

A solution of the set of two equations, involving differential equation (60) and integro-differential equation (62), allows one to find the time-dependence of the values *W* and $\alpha$.

Note that since the impact process is of short duration, then in the integrals entering in (62)

63 $$\begin{aligned} \ni _\gamma \left( - \frac{t}{t_j}\right) \approx \frac{t^{\gamma -1}}{t_j^\gamma \Gamma (\gamma )} \quad (j=1,2). \end{aligned}$$

Equations (60) and (62) are subjected to the initial conditions (26).

### Solution of the problem in the case of neglecting the extension of the beam’s middle surface

If the extension of the middle surface is excluded from consideration, then the set of the governing equations (60) and (62) with due account for (63) is reduced to the following:

64 $$\begin{aligned} m\dot{W} + m\ddot{\alpha }= & {} - k_1 \left[ \alpha ^{3/2} - \Delta _\gamma \int _0^t (t-t')^{\gamma -1} \alpha ^{3/2}(t') dt' \right] , \end{aligned}$$

65 $$\begin{aligned} \dot{W} + \frac{g}{m}\, W= & {} - \ddot{\alpha }, \end{aligned}$$

where

$$\begin{aligned} \Delta _\gamma =\frac{1}{\Gamma (\gamma )}\left( \frac{e_1}{t_1^\gamma }+\frac{e_2}{t_2^\gamma }\right) . \end{aligned}$$

The solution of (65) could be constructed in the form

66 $$\begin{aligned} W = C(t) e^{-\frac{g}{m}\,t}, \end{aligned}$$

where the function *C*(*t*) could be found using the method of variation of an arbitrary constant, resulting in

67 $$\begin{aligned} m \dot{W} + m \ddot{\alpha }=g \dot{\alpha } - \frac{g^2}{m}\, \alpha - g V_0. \end{aligned}$$

Substituting (67) in (62), we arrive at the governing integro-differential equation

68 $$\begin{aligned} \dot{\alpha } - \frac{g}{m}\, \alpha =- f_1 \left[ \alpha ^{3/2} -\Delta _\gamma \int _0^t (t-t')^{\gamma -1} \alpha ^{3/2}(t') dt' \right] + V_0 \end{aligned}$$

subjected to the initial conditions

69 $$\begin{aligned} \dot{\alpha }|_{t=0}=V_0, \quad \alpha |_{t=0}=0, \end{aligned}$$

where $$f_1=\frac{k_1}{g}=\frac{k_1}{2\rho FG_\infty ^{(2)}}$$.

A solution of (68) could be found using the method of successive approximations. Thus, a particular solution of (68) neglecting its terms in square brackets has the form

70 $$\begin{aligned} \alpha (t)= \frac{m}{g}\,V_0\left( e^{\frac{g}{m}\,t}-1\right) . \end{aligned}$$

Expanding exponent

71 $$\begin{aligned} e^{\frac{g}{m}\,t}\approx 1+\frac{g}{m}\,t , \end{aligned}$$

and substituting (71) in (70) yield

72 $$\begin{aligned} \alpha \approx V_0 t. \end{aligned}$$

Now substituting (72) in the right-hand side of (68), we could find the general solution of the obtained nonhomogeneous equation in the form

73 $$\begin{aligned} \alpha = C_1(t) e^{\frac{g}{m}\, t}- V_0 \,\frac{m}{g}, \end{aligned}$$

where the function $C_1(t)$ could be found by the method of variation of an arbitrary constant

74 $$\begin{aligned} C_1(t) =- f_1 V_0^{3/2} \left( \frac{2}{5}\,t^{5/2} - \Delta _\gamma \int _0^t dt'' \left[ \int _0^{t''} (t''-t')^{\gamma -1} {t'}^{3/2}(t') dt'\right] \right) + C_0, \end{aligned}$$

where $C_0$ is the constant of integration, resulting in the following final solution:

75 $$\begin{aligned} \alpha= & {} \frac{m}{g}\,V_0\left( e^{\frac{g}{m}\,t}-1\right) \nonumber \\&- f_1 V_0^{3/2}\left( \frac{2}{5}\, t^{5/2} -\Delta _\gamma \int _0^t dt'' \left[ \int _0^{t''} (t''-t')^{\gamma -1} {t'}^{3/2}(t') dt'\right] \right) e^{\frac{g}{m}\, t} . \end{aligned}$$

Considering (71), solution (75) for two limiting cases is reduced to the following:

at $$\gamma =0$$

76 $$\begin{aligned} \alpha =V_0 t- \frac{2}{5}\, f_1 V_0^{3/2} t^{5/2} e^{\frac{g}{m}\, t} , \end{aligned}$$

and at $$\gamma =1$$

77 $$\begin{aligned} \alpha =V_0 t- \frac{2}{5}\, f_1 V_0^{3/2}\, t^{5/2} e^{\frac{g}{m}\, t} + \frac{4}{35}\, \Delta f_1 V_0^{3/2}t^{7/2} e^{\frac{g}{m}\, t} , \end{aligned}$$

where

$$\begin{aligned} \Delta =\frac{e_1}{t_1}+\frac{e_2}{t_2}. \end{aligned}$$

Relationships (76) and (77) allow one to estimate for the limiting cases the time of impactor’s rebound from the target $t_\mathrm{reb}$ and the time $t_\mathrm{max}$ at which the indentation attains its maximal magnitude $\alpha _\mathrm{max}$, i.e., at $\gamma =0$

78 $$\begin{aligned} t^0_\mathrm{reb}= & {} \left( \frac{5}{2f_1}\,V_0^{-1/2} \right) ^{2/3}, \end{aligned}$$

79 $$\begin{aligned} t^0_\mathrm{max}= & {} \left( \frac{1}{f_1}\,V_0^{-1/2} \right) ^{2/3}, \end{aligned}$$

80 $$\begin{aligned} \alpha ^0_\mathrm{max}= \, V_0t^0_\mathrm{max}-\frac{2}{5}\;f_1 V_0^{3/2} \left( t^0_\mathrm{max}\right) ^{5/2} = \frac{3}{5}\,V_0^{2/3} f_1^{-2/3}, \end{aligned}$$

and at $$\gamma =1$$

81 $$\begin{aligned} t^1_\mathrm{reb}= \, t^0_\mathrm{reb}+ \frac{4}{21}\,\Delta \,\left( t^0_\mathrm{reb}\right) ^{2} , \end{aligned}$$

82 $$\begin{aligned} t^1_\mathrm{max}= \, t ^0_\mathrm{max}+\frac{4}{15}\, \Delta \, \left( t^0_\mathrm{max}\right) ^{2}, \end{aligned}$$

83 $$\begin{aligned} \alpha ^1_\mathrm{max}= \, \alpha ^0_\mathrm{max}+ \frac{4}{35}\,\Delta \,f_1 V_0^{3/2} \left( t^0_\mathrm{max}\right) ^{7/2}. \end{aligned}$$

Reference to formulas (78)–(83) shows that the increase in the parameter $\gamma$ from 0 to 1 results in the increase of the duration of contact between the impactor and the viscoelastic target, and this increase rises with the increase in the defects of moduli $e_1$ and $e_2$ and with the decrease in the relaxation times $t_1$ and $t_2$. Moreover, within the variation of the parameter $\gamma$ from 0 to 1, the maximal magnitude of the value $\alpha$, as well as the time, at which the indentation attains its maximum, increase. All enumerated peculiarities are governed by the increase in viscosity of the material, from which the viscoelastic beam is made of, with the increase of the fractional parameter $\gamma$.

### Solution of the problem in the case of considering the extension of the beam’s middle surface

#### Elastic target

If the term $\alpha ^{1/2} \dot{W}$ is omitted in (58) due to its small magnitude, i.e. the inertia of the contact domain is neglected, then this equation for an elastic beam is reduced to

84 $$\begin{aligned} W=\frac{P(\alpha )}{e\alpha +g}. \end{aligned}$$

Substituting (84) in (59), we obtain the equation for determining $\alpha (t)$

85 $$\begin{aligned} \ddot{\alpha } +\frac{d}{d\alpha }\left( \frac{P(\alpha )}{e\alpha +g}\right) \dot{\alpha } =-\frac{1}{m}\, P(\alpha ), \end{aligned}$$

or, after the substitution $A=\dot{\alpha }$,

86 $$\begin{aligned} A\, \frac{d A}{d \alpha } +\frac{d}{d\alpha }\left( \frac{P(\alpha )}{e\alpha +g}\right) A =-\frac{1}{m}\, P(\alpha ). \end{aligned}$$

To find the solution of (86), it is necessary to use the initial condition

87 $$\begin{aligned} A|_{\alpha =0}=V_0. \end{aligned}$$

The governing Eq. (85) differs from the corresponding equation presented in Vershinin (2014) not only by its coefficients (due to the fact that an incorrect formula was used for transverse deformation) but by its structure as well, namely: the multiplier $\dot{\alpha }$ was missed out in the second term, though the inertia of the contact zone was not neglected in the cited paper (Vershinin 2014).

Equation (86) could be rewritten in the form

88 $$\begin{aligned} A\, \frac{d A}{d \alpha } +k_1\; \frac{e\alpha ^{3/2}+3/2\,g\alpha ^{1/2} }{(e\alpha +g)^2}\; A =-\frac{k_1}{m}\, \alpha ^{3/2}. \end{aligned}$$

If we neglect the term $e\alpha$ with respect to *g* ($\alpha$ is small) in (88), then it is reduced to

89 $$\begin{aligned} A\, \frac{d A}{d \alpha } +\frac{3}{2}\,f_1\alpha ^{1/2} A +f_2\; \alpha ^{3/2} A =-\frac{k_1}{m}\, \alpha ^{3/2}, \end{aligned}$$

where

$$\begin{aligned} f_2=\frac{k_1e}{g^2}=f_1\, \frac{{G_\infty ^{(1)} }^2}{{G_\infty ^{(2)} }^2}\, \frac{1}{\nu _\infty h}. \end{aligned}$$

The solution of (89) can be constructed analytically in terms of a series

90 $$\begin{aligned} A=V_0+\sum \limits _{i=1}^\infty a_i\alpha ^{(2i+1)/2}+\sum \limits _{i=1}^\infty b_i\alpha ^{i}, \end{aligned}$$

where

$$\begin{aligned} a_1= & {} -f_1, \quad a_2=-\frac{2}{5}\;\frac{k_1}{V_0m} -\frac{2}{5}\, f_2, \quad a_3=a_4=0, \quad a_5=\frac{5}{11}\,\frac{a_1^2(a_2+f_2)}{V_0^2},\\ a_6= \, \frac{5}{104}\,\frac{a_1\left( a_2+\frac{2}{5}\, f_2\right) (21a_2+2f_2)}{V_0^2}, \quad a_7=\frac{1}{6}\, \frac{a_2\left( a_2+\frac{2}{5}\, f_2\right) (3a_2+\frac{2}{5}\,f_2)}{V_0^2},\\ b_1= \, b_2=b_3=b_6=0, \quad b_4=-\frac{5}{8}\, \frac{a_1\left( a_2+\frac{2}{5}\, f_2\right) }{V_0},\\ b_5= & {} -\frac{1}{2}\, \frac{a_2\left( a_2+\frac{2}{5}\, f_2\right) }{V_0}, \quad b_7=-\frac{5}{14}\, \frac{a_1^3\left( a_2+\frac{2}{5}\, f_2\right) }{V_0^3}. \end{aligned}$$

From (90) it is seen that all coefficients $a_i$ ($i\ge 5$) and $b_i$ ($i \ge 4$) are expressed in terms of the coefficients $a_1$ and $a_2$, which are defined by three different processes being caused by the shock interaction. The coefficient $a_1$ is responsible for the dynamic processes arising in the beam during the propagation of shear wave, but the coefficient $a_2$ answers for the quasistatic process occurring at local bearing of the material due to Hertz’s theory and for the dynamic processes arising in the beam during the propagation of the longitudinal wave.

When $g = 0$, which is realized at an infinitely large speed of the shear wave propagation, the solution (90) for small $\alpha$ goes over into the quasi-static solution obtained by Timoshenko (1934) for the Bernoulli–Euler beam.

If we put $f_2 = 0$ in (90) in order to neglect membrane effects at $e = 0$, then the series (90) is the solution to equation

91 $$\begin{aligned} A\, \frac{d A}{d \alpha } +\frac{3}{2}\,f_1\alpha ^{1/2} A = -\frac{k_1}{m}\, \alpha ^{3/2}, \end{aligned}$$

which was presented in Rossikhin and Shitikova (2007a).

#### Viscoelastic target

Neglecting the inertia of the contact domain in (60), expressing $\dot{W}+\ddot{\alpha }$ from (62) with due account for (63), and substituting the resulting expression in (60) yield

92 $$\begin{aligned} W = \frac{f_1}{\frac{g}{m} + \frac{e}{ m}\,\alpha } \left[ \alpha ^{3/2} - \Delta _\gamma \int _0^t (t-t')^{\gamma -1} \alpha ^{3/2}(t') dt' \right] . \end{aligned}$$

Considering that $\alpha$ is a small value, expanding $\left( \frac{g}{m} + \frac{e}{ m}\,\alpha \right) ^{-1}$ into a Taylor series, and limiting ourselves by two terms, Eq. (92) is reduced to

93 $$\begin{aligned} W = f_1 \left[ \alpha ^{3/2} - \Delta _\gamma \int _0^t \alpha ^{3/2}(t') dt' \right] \frac{m}{g}\left( 1- \frac{e}{ g}\,\alpha \right) . \end{aligned}$$

Now substituting (72) in the right-hand side of (93) and putting $\gamma = 1$, we could find

94 $$\begin{aligned} W = f_1V_0 ^{3/2} t^{3/2} \left( 1- \frac{2}{5}\, \Delta t- \frac{e}{ g}\, V_0 t\right) . \end{aligned}$$

Substituting (94) in (65) and integrating twice yield

95 $$\begin{aligned} \alpha = V_0t-\frac{2}{5}\,f_1 V_0 ^{3/2} t^{5/2} +\frac{2}{7}\, f_1 V_0 ^{3/2} \left( \frac{2}{5}\, \Delta + \frac{e}{ g}\, V_0 \right) t^{7/2}. \end{aligned}$$

Putting $\gamma = 0$ in (94) and (95), we could find the solution for this limiting case

96 $$\begin{aligned} W= \, f_1 V_0 ^{3/2} t^{3/2} \left( 1- \frac{e}{ g}\, V_0 t\right) , \end{aligned}$$

97 $$\begin{aligned} \alpha= \, V_0t-\frac{2}{5}\,f_1 V_0 ^{3/2} t^{5/2} +\frac{2}{7}\, f_1\,\frac{e}{g}\, V_0 ^{5/2} t^{7/2}. \end{aligned}$$

Relationships (95) and (97) allow one to estimate for the limiting cases the time of impactor’s rebound from the target $t_\mathrm{reb}^\mathrm{ex}$ and the time $t_\mathrm{max}^\mathrm{ex}$ at which the indentation attains its maximal magnitude $\alpha _\mathrm{max}^\mathrm{ex}$ with due account for extension of the beam’s middle surface, i.e., at $\gamma = 0$

98 $$\begin{aligned} t^{0\,\mathrm ex}_\mathrm{reb}= \, t^{0}_\mathrm{reb}+ \frac{10}{21}\,V_0 \, \frac{e}{g}\, \left( t^{0}_\mathrm{reb}\right) ^2, \end{aligned}$$

99 $$\begin{aligned} t^{0\,\mathrm ex}_\mathrm{max} = \, t^{0}_\mathrm{max}+ \frac{2}{3}\,V_0 \frac{e}{g}\,\left( t^{0}_\mathrm{max}\right) ^2, \end{aligned}$$

100 $$\begin{aligned} \alpha ^{0\,\mathrm ex}_\mathrm{max} = \, \alpha ^{0}_\mathrm{max}+\frac{2}{7}\, f_1\,\frac{e}{g}\,V_0^{5/2} \left( t^{0}_\mathrm{max}\right) ^{7/2}, \end{aligned}$$

and at $$\gamma = 1$$

101 $$\begin{aligned} t^{1\,\mathrm ex}_\mathrm{reb} = \, t^1_\mathrm{reb} + \frac{10}{21}\,V_0 \, \frac{e}{g}\, \left( t^{0}_\mathrm{reb}\right) ^2, \end{aligned}$$

102 $$\begin{aligned} t^{1\,\mathrm ex} _\mathrm{max} = \, t^{1}_\mathrm{max}+ \frac{2}{3}\,V_0 \frac{e}{g}\,\left( t^{0}_\mathrm{max}\right) ^2, \end{aligned}$$

103 $$\begin{aligned} \alpha ^{1\,\mathrm ex}_\mathrm{max} = & {} \alpha ^1_\mathrm{max}+ \frac{2}{7}\,f_1\,\frac{e}{g}\, V_0^{5/2} \left( t^{0}_\mathrm{max}\right) ^{7/2}. \end{aligned}$$

Comparison of formulas (98)–(103) with the corresponding formulas in (78)–(83) shows that the account for extension of the target’s middle surface results in the increase of the duration of contact between the impactor and the target, the maximal magnitude of the value $\alpha$, as well as the time, at which the indentation attains its maximum, and this increase rises with the increase in the coefficient *e* / *g* governing the extension of the beam's middle surface.

#### Numerical example

For numerical analysis it is convenient to rewrite formulas (78)–(83) and (98)–(103) in the dimensionless form:

the case without considering the middle surface extension at $\gamma = 0$

104 $$\begin{aligned} {t^0_\mathrm{reb}}^*= \, \frac{t^0_\mathrm{reb}}{t^0_\mathrm{max}}=\left( \frac{5}{2} \right) ^{2/3}, \end{aligned}$$

105 $$\begin{aligned} {t^0_\mathrm{max}}^*= \,\frac{t^0_\mathrm{max}}{t^0_\mathrm{max}}=1, \end{aligned}$$

106 $$\begin{aligned} {\alpha ^0_\mathrm{max}}^*= \, \frac{\alpha ^0_\mathrm{max}}{V_0 t^0_\mathrm{max}}= \frac{3}{5}, \end{aligned}$$

and at $$\gamma = 1$$

107 $$\begin{aligned} {t^1_\mathrm{reb}}^*= \, \frac{t^1_\mathrm{reb}}{t^0_\mathrm{max}}= \left( \frac{5}{2}\right) ^{2/3} \left[ 1 + \frac{4}{21}\;\chi _1 \left( \frac{5}{2} \right) ^{2/3}\right] , \end{aligned}$$

108 $$\begin{aligned} {t ^1_\mathrm{max}}^*= \, \frac{t^1_\mathrm{max}}{t^0_\mathrm{max}}=1+\frac{4}{15}\,\chi _1, \end{aligned}$$

109 $$\begin{aligned} {\alpha ^1_\mathrm{max}}^*= \, \frac{\alpha ^1_\mathrm{max}}{V_0t^0_\mathrm{max}}=\frac{3}{5}+ \frac{4}{35}\,\chi _1; \end{aligned}$$

the case with due account for extension of the beam’s middle surface at $\gamma = 0$

110 $$\begin{aligned} {t^{0\,\mathrm ex}_\mathrm{reb}}^*= \, \frac{t^{0\,\mathrm ex}_\mathrm{reb}}{t^0_\mathrm{max}} =\left( \frac{5}{2}\right) ^{2/3} \left[ 1+ \frac{10}{21}\;\chi _2 \left( \frac{5}{2} \right) ^{2/3}\right] , \end{aligned}$$

111 $$\begin{aligned} {t^{0\,\mathrm ex}_\mathrm{max}}^*= \, \frac{t^{0\,\mathrm ex}_\mathrm{max}}{t^0_\mathrm{max}}=1+\frac{2}{3}\,\chi _2, \end{aligned}$$

112 $$\begin{aligned} {\alpha ^{0\,\mathrm ex}_\mathrm{max}}^*= \, \frac{\alpha ^{0\,\mathrm ex}_\mathrm{max}}{V_0t^0_\mathrm{max}} =\frac{3}{5}+ \frac{2}{7}\,\chi _2, \end{aligned}$$

and at $$\gamma = 1$$

113 $$\begin{aligned} {t^{1\,\mathrm ex}_\mathrm{reb}}^*= \, \frac{t^{1\,\mathrm ex}_\mathrm{reb}}{t^0_\mathrm{max}} =\left( \frac{5}{2}\right) ^{2/3} \left[ 1+ \frac{4}{21}\;\chi _1 \left( \frac{5}{2} \right) ^{2/3}\right] + \frac{10}{21} \,\chi _2\left( \frac{5}{2}\right) ^{4/3}, \end{aligned}$$

114 $$\begin{aligned} {t^{1\,\mathrm ex} _\mathrm{max}}^*= \, \frac{t^{1\,\mathrm ex}_\mathrm{max}}{t^0_\mathrm{max}}=1+\frac{4}{15}\,\chi _1 +\frac{2}{3}\,\chi _2, \end{aligned}$$

115 $$\begin{aligned} {\alpha ^{1\,\mathrm ex}_\mathrm{max}}^*= \, \frac{\alpha ^{1\,\mathrm ex}_\mathrm{max}}{V_0t^0_\mathrm{max}} =\frac{3}{5}+\frac{4}{35}\,\chi _1+ \frac{2}{7}\,\chi _2, \end{aligned}$$

where $\chi _1= \Delta t^0_\mathrm{max}$ and $\chi _2= V_0\,eg^{-1}t^0_\mathrm{max}$ are dimensionless parameters, and all dimensionless values are marked by asterisk.

**Fig. 2:**
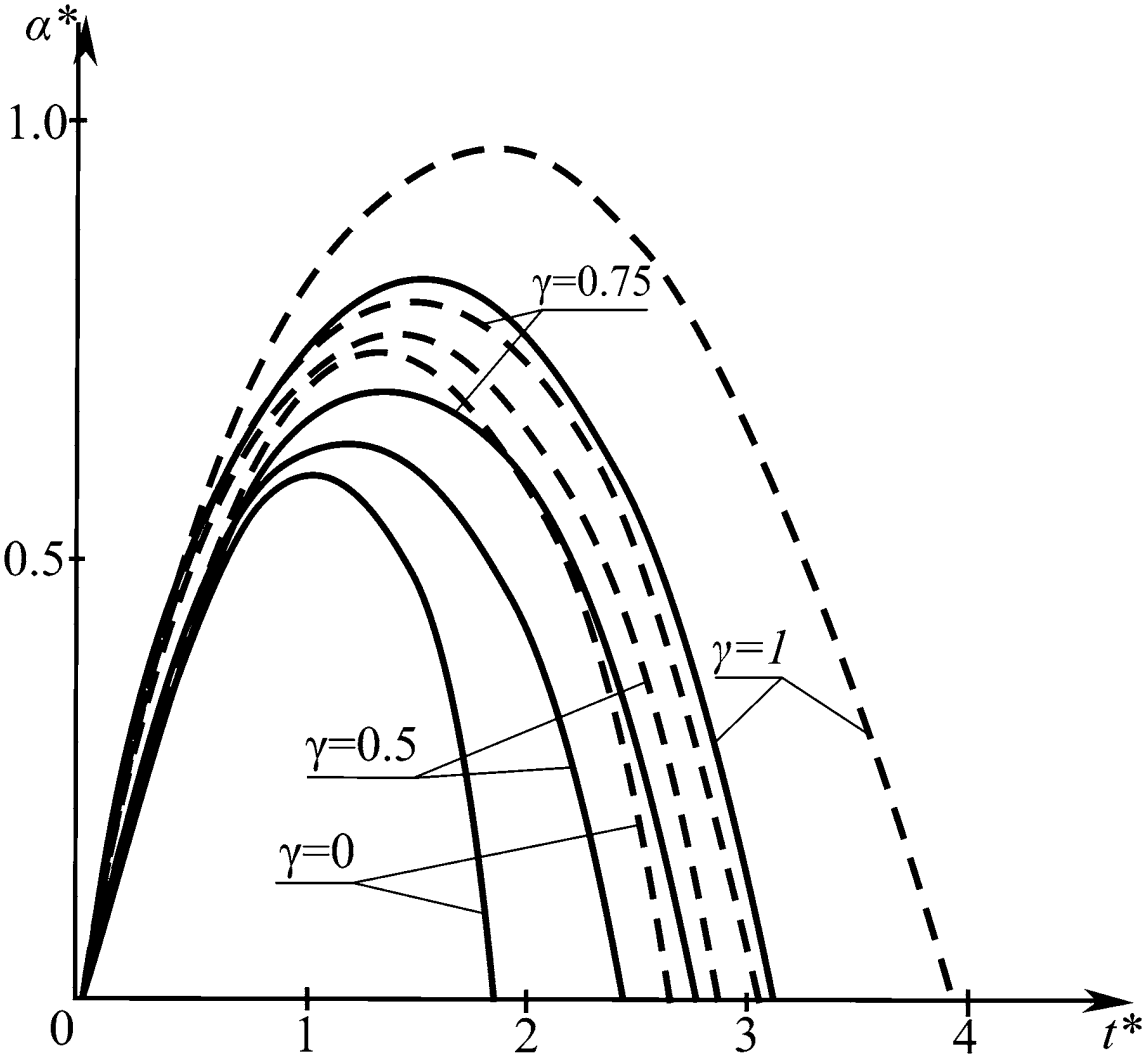
The dimensionless time dependence of the dimensionless value characterizing the local bearing of impactor and target materials

The dimensionless time $t^*= t\left( t^0_\mathrm{max}\right) ^{-1}$ dependence of the dimensionless value $\alpha ^*= \alpha \left( V_0 t^0_\mathrm{max}\right) ^{-1}$ characterizing the local bearing of impactor and target materials for different values of the fractional parameter $\gamma$, which are indicated by figures near the corresponding curves, is shown in Fig. 2. Solid curves are calculated without considering the extension of the target middle surface, while dashed lines correspond to the case with due account for middle surface extension at $\chi _1=2$ and $\chi _2=0.5$.

The character of curves behaviour in Fig. 2 verifies conclusions made on the basis of the approximate calculations.

## Conclusion

The main goal of this paper is to bring to light the physical sense of the fractional parameter in problems on impact, since one and the same question arises very often, namely: Why is it needed to introduce a fractional derivative in problems of mechanics? The authors have tried to answer this question at least for the problems of impact by connecting the fractional parameter with the changes in microstructure of beam’s material within the contact domain. For this purpose we have assumed that viscoelastic features of the beam outward the contact zone is determined by the standard linear solid with ordinary derivatives, while the contact force is also viscoelastic and its features are governed by the standard linear solid model with fractional derivatives, in so doing relaxed and non-relaxed moduli and relaxation and retardation times coincide with the corresponding moduli and times for the viscoelastic medium out of the contact zone, and the fractional parameter varies from zero till unit controlling the viscosity within the contact domain. This is connected with the fact that during the low-velocity impact there could occur decrosslinking within the domain of the contact of the beam with the sphere, resulting in more freely displacements of molecules with respect to each other, and finally in the decrease of the beam material viscosity in the contact zone without discontinuity of the target medium within this zone.
